# Multi-Scale Functional Network Connectivity Mediates The Association Between Bullying Exposure and Distressing Psychotic-Like Experiences in Healthy Adolescents

**DOI:** 10.1192/j.eurpsy.2025.302

**Published:** 2025-08-26

**Authors:** P. Andrés Camazón, R. Ballem, J. Chen, V. Calhoun, J. Galvañ, L. Lopez, J. C. Pedrosa, B. Sáez, M. Alonso, J. Suárez, N. Cruz, M. Gonzalo, M. R. Lopez, B. Arribas, A. Iraji, C. M Díaz-Caneja

**Affiliations:** 1 Institute of Psychiatry and Mental Health, Hospital General Universitario Gregorio Marañón, IiSGM, CIBERSAM, ISCIII, School of Medicine, Universidad Complutense, Madrid, Spain, Madrid, Spain; 2 Translational Research in Neuroimaging and Data Science (Georgia State University, Georgia Institute of Technology, Emory University), Atlanta, Georgia, United States., Atlanta, United States; 3 Institute of Psychiatry and Mental Health, Hospital General Universitario Gregorio Marañón, Madrid, Spain

## Abstract

**Introduction:**

Bullying, deliberate aggression by a peer or group of peers in a power imbalance that favors the aggressor, is a frequent and preventable traumatic event during adolescence (Abregú-Crespo R et al. The Lancet Child & Adolescent Health 2024; 8:122–134). Mitigating its impact could be a viable strategy for psychosis prevention (Fraguas, D. et al. JAMA Pediatrics 2021; 175, 44–55). A better understanding of its influence on brain development during adolescence could be crucial for implementing effective interventions.

**Objectives:**

To study the relationship between bullying exposure (BE), distressing psychotic-like experiences (DPLEs), and multi-scale functional network connectivity (msFNC) in the developing brains of 12-year-old adolescents.

**Methods:**

We used cross-sectional data from 12-year-old adolescents from the Adolescent Brain Cognitive Development Study, which recruited a representative sample of healthy adolescents from across the United States. We fitted a linear mixed model to predict DPLEs (Prodromal Questionnaire-Brief Child Version) with BE (Peer Experience Questionnaire) as a predictor (n=10,388). We analyzed functional magnetic resonance imaging data with reference-informed independent component analysis and a canonical and replicable multi-scale intrinsic connectivity network template to extract whole-brain 5460 msFNC features (Iraji, A. et al. Hum Brain Mapp 2023 44, 5729–5748). We fitted 5460 linear mixed models to predict DPLEs with BE as a predictor and analyzed the mediation effect of each of the 5460 FNC features (n=5,409). All models were fitted with family and site as random effects, adjusted for covariates (age, sex, race, ethnicity, pubertal development, and family income), and corrected for multiple comparisons.

**Results:**

BE was significantly associated with DPLEs (β=0.39, CI[0.37, 0.41], t(10362) = 41.00, p<.001, R^2^=0.36, p<.000) (**Fig.1**). DPLEs were associated with msFNC predominantly between cerebellar, paralimbic, somatomotor, insulotemporal, frontal, temporoparietal, and central executive networks (**Fig.2**). The association between DPLEs and BE was primarily mediated by msFNC between the paralimbic, somatomotor, insulotemporal, frontal, and temporoparietal networks (**Fig.3**).

**Image 1:**

**Figure:**
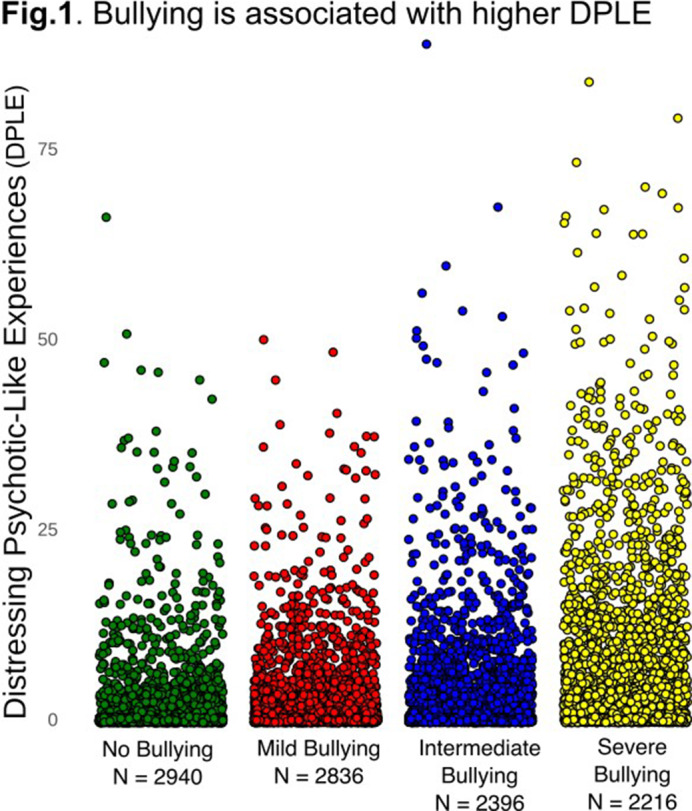

**Image 2:**

**Figure:**
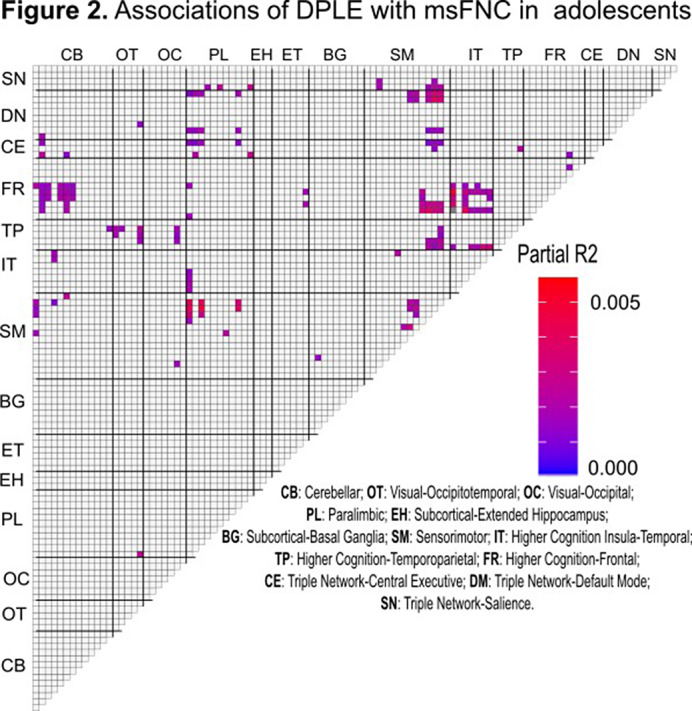

**Image 3:**

**Figure:**
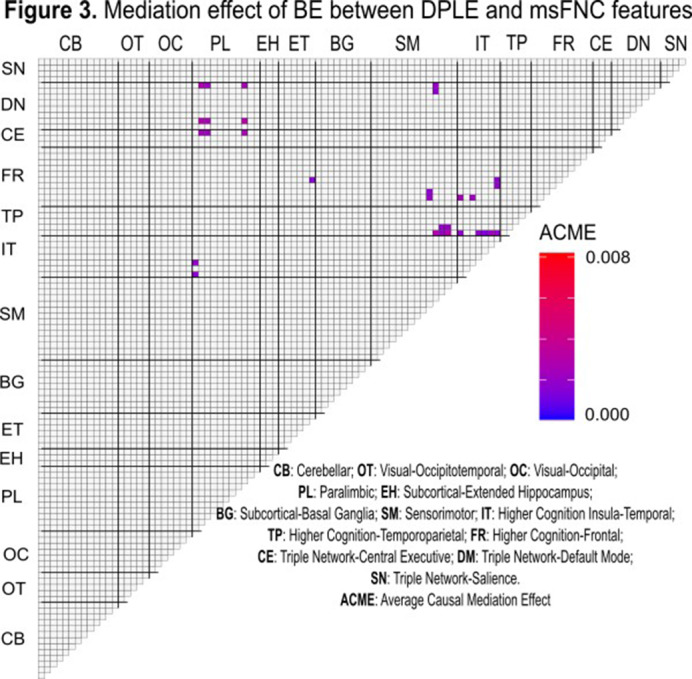

**Conclusions:**

Bullying exposure may represent a modifiable risk factor for the development of DPLEs during adolescence. It may influence DPLEs through its effects on relevant functional brain networks. The implementation of targeted interventions to prevent BE during adolescence could serve as a viable strategy to mitigate potential functional brain alterations and reduce the risk of psychosis.

**Disclosure of Interest:**

None Declared

